# A “double fistula” with *Mycobacterium avium* complex lung disease

**DOI:** 10.1002/rcr2.809

**Published:** 2021-07-05

**Authors:** Ahel El Haj Chehade, Ibrahim Raphael, Brent Brown, Tony Abdo

**Affiliations:** ^1^ Department of Pulmonary, Critical Care and Sleep Medicine The University of Oklahoma Health and Sciences Center Oklahoma City OK USA

**Keywords:** COPD, fistula, iatrogenic, *Mycobacterium avium* complex

## Abstract

We present a patient with a history of cavitary mycobacterial lung infection and chronic obstructive pulmonary disease (COPD) who developed two connected fistulas post thoracentesis, an alveolo‐pleural and a pleuro‐cutaneous fistula, leading to continuous air leak from a stoma on his back.

## Graphical abstract

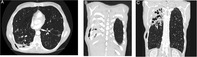

## Video

**Figure 1:**
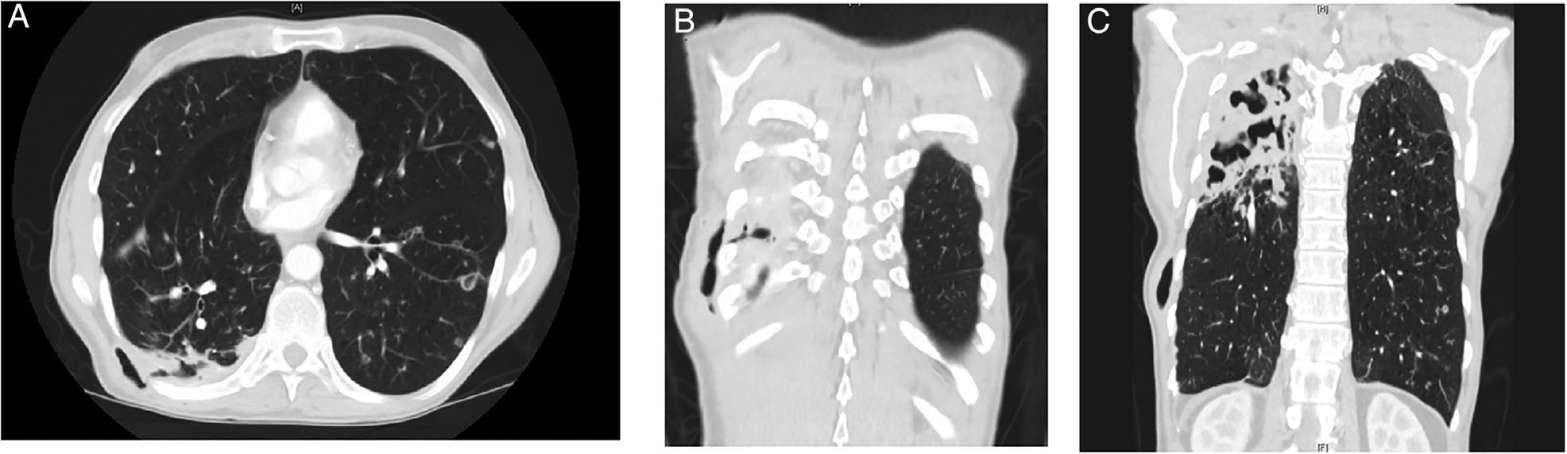
(A, B) Fistulous tract between the lung parenchyma, the pleura, and the skin. (C) Subcutaneous air and necrotizing cavitations in the right upper lobe.

A 60‐year‐old male presented to the pulmonary clinic with air leaking from his back that started three weeks prior to presentation (Video S1, Part 1). The patient has a history of severe chronic obstructive pulmonary disease, right‐sided secondary spontaneous pneumothorax status post video‐assisted thoracoscopic surgery (VATS) pleurodesis and bullectomy, and a four‐month history of pulmonary *Mycobacterium avium* complex (MAC) infection on antimicrobial therapy. One month prior, he was hospitalized for pneumonia at another facility and had a diagnostic right‐sided thoracentesis. He reported that the thoracentesis site never healed; it was leaking fluid first and then a week later it started leaking air. The patient was admitted to the hospital. Computed tomography of the chest showed a “double fistula” consisting of alveolo‐pleural and pleuro‐cutaneous fistulas (Fig. 1A, B) and right upper lobe cavitary lung disease secondary to his MAC infection (Fig. 1C). A bronchoscopic evaluation with methylene blue injection into the fistula tract failed to locate the fistula's bronchial end. Biopsies from the fistula did not grow any microorganisms and were negative for malignancy. Given his advanced lung disease, he was considered a poor candidate for a lobectomy with fistula closure. Follow‐up after four months in the clinic revealed near‐complete healing of the pleuro‐cutaneous fistula stoma without the patient developing any clinical sign or symptoms of tension pneumothorax (Video S1, Part 2).

### Disclosure Statement

Appropriate written informed consent was obtained for publication of this case report and accompanying images and video.

## Supporting information


**Video S1.** Part 1: Initial evaluation of our 60‐year‐old patient showing air leaking from his back that started three weeks prior to presentation. Part 2: Four‐month follow‐up showing near‐complete healing of the pleuro‐cutaneous fistula stoma without the patient developing any clinical sign or symptoms of tension pneumothorax.

